# Radiological Determinants of Thromboembolic Events in COVID-19 Pneumonia: A Retrospective Study

**DOI:** 10.7759/cureus.27736

**Published:** 2022-08-06

**Authors:** Mohd Ghadeeb, Ali M Aljazzar, Rahaf A Amri, Abdulrahman F Alamoudi, Atheer A Alghamdi, Tariq S Al-Shairbeeny, Ali A Alnasser, Ahmed K Alsenan

**Affiliations:** 1 Radiology, King Fahad Hospital, Al Hofuf, SAU; 2 Radiology, Dammam Medical Center, Dammam, SAU; 3 General Medicine, Jazan University, Jazan, SAU; 4 General Medicine, AlMaarefa University, Ad Diriyah, SAU; 5 General Medicine, Albaha University, Al Baha, SAU; 6 General Medicine, Abqaiq General Hospital, Abqaiq, SAU; 7 General Medicine, King Fahad University Hospital, Al Khobar, SAU

**Keywords:** pneumonia, deep venous thrombosis, pulmonary embolism, computed tomography, covid-19

## Abstract

Background

It has been established that patients with COVID-19 pneumonia are more vulnerable to developing thromboembolic complications. Computed tomography (CT) scan of the chest is an essential investigation modality in patients with COVID-19 pneumonia and has an important role in the diagnosis and identification of complications.

Methods

A retrospective observational study was conducted on patients admitted with COVID-19 pneumonia who underwent CT scans of the chest. The data regarding demographic information, clinical information, and CT findings were collected from electronic health records. Multivariable regression analysis was used to identify the independent factors associated with thromboembolic complications.

Results

The study included a total of 276 patients, including 178 (64.5%) men and 98 (35.5%) women. In total, 64 patients were found to have thromboembolic events, yielding a complication rate of 23.2%. Multivariable logistic regression revealed that patients aged 51-65 years (Odds ratio [OR] = 8.9; 95% confidence interval [CI]: 3.0-26.5) and >65 years (OR = 18.7; 95% CI: 7.6-46.1) had a higher likelihood of having thromboembolic complications compared to those aged 18-35 years. Further, the crazy-paving appearance of opacity was identified as an independent factor associated with thromboembolic events (OR = 14.2; 95% CI: 6.9-29.4). Further, patients with severe pulmonary parenchymal involvement were 30 times (OR = 30.6; 95% CI: 9.8-95.5) more likely to have thromboembolic complications compared with those having mild involvement.

Conclusions

The radiological findings on the CT scan of the chest can provide crucial prognostic information for patients with COVID-19 in terms of thromboembolic events. Clinicians need to keep a high index of suspicion for pulmonary embolism and deep venous thrombosis when they encounter patients with crazy-paving opacity appearances on CT scans, particularly among patients with severe parenchymal involvement.

## Introduction

The coronavirus disease 2019 (COVID-19), caused by severe acute respiratory syndrome coronavirus 2 (SARS-CoV-2), predominantly affects the respiratory tract and may cause acute respiratory failure. However, early in the course of the COVID-19 pandemic, it became evident that COVID-19 is a complex multisystem disorder [1]. Several studies have demonstrated the increased risk of thromboembolic complications, particularly among critically ill patients [2,3]. While the exact pathogenesis of hypercoagulability is incompletely understood, contributing factors can be thought of in terms of Virchow’s triad, namely, endothelial injury, stasis, and hypercoagulable state. For instance, derangement in some of the coagulation factors has been reported, including elevated factor VIII and fibrinogen [4]. Thromboembolic manifestations have a wide spectrum and vary significantly among different patients. These include venous thromboembolic events, arterial events, and microvascular thrombosis [5,6].

The diagnosis of venous thromboembolic events, including deep venous thrombosis and pulmonary embolism, can be challenging due to overlapping clinical and laboratory features. Computed tomography pulmonary angiography (CTPA) is the investigation modality of choice to detect pulmonary embolisms. Further, computed tomography (CT) has additional roles in the setting of COVID-19. These include the evaluation of the progression of pulmonary parenchymal involvement and prediction of the prognosis of the disease [7,8]. However, limited studies have investigated the association between the CT findings and the risk of thromboembolic complications. Therefore, this study aims to evaluate the prevalence of such complications and potential CT features, which may serve as independent factors associated with thromboembolic complications.

## Materials and methods

Study design and setting

After obtaining approval from the Ethics Committee of the Ministry of Health, a retrospective study to investigate the determinants of thromboembolic complications in patients with COVID-19 pneumonia was conducted. All methods were carried out in accordance with relevant guidelines and regulations. The need for informed consent was waived considering the retrospective nature of the study. The study was conducted at King Fahad Hospital, Al Hofuf, one of the largest tertiary public hospitals in the Eastern Province of Saudi Arabia. It has a capacity of 500 beds and has over 100,000 visits to the emergency department annually.

Study population

The hospital information system was utilized to obtain the list of all hospitalized patients with COVID-19 pneumonia between January 2021 and June 2022. Eligible patients were adults with positive results on reverse transcriptase-polymerase chain reaction (RT-PCR) for SARS-CoV-2 and underwent a CT scan of the chest. Exclusion criteria were as follows: age below 18 years and pregnant patients.

Data collection

A data collection form was used to collect the data from the electronic health records. The exposure variables included demographic, clinical, and radiological information. The demographic data included age, gender, smoking status, and body mass index (BMI). The clinical information included the duration of symptoms and the need for mechanical ventilation. The collected radiological findings on the CT scan included the pattern, distribution, and extent of opacities. Also, additional features like pleural effusion, lymphadenopathy, and air bronchogram were collected. The outcome variable was the need for mechanical ventilation during the hospitalization. The collected data were reviewed for completeness and accuracy by the principal investigator. The severity of parenchymal involvement was quantified according to the extent of involvement of each of the six lung zones. Each zone was assigned a score (score 0: 0% involvement; score 1: <25% involvement; score 2: 25%-50% involvement; score 3: 50%-75% involvement; and score 4: 75%-100% involvement). The total score was then calculated, and the severity was classified into mild (total score < 7), moderate (total score: 7-17), and severe (total score > 17).

Statistical analysis

The data were compiled using Microsoft Excel 2019 (Microsoft Corp., Redmond, Washington) and were analyzed using the IBM SPSS (Statistical Package for the Social Sciences) software (IBM Corp., Armonk, NY). Categorical variables, presented as percentages and frequency distribution, were compared using the chi-squared or Fisher’s exact tests. Figures were used to summarize the radiological findings of the CT scan. Multivariable binary logistic regression analysis was conducted to identify the independent predictors of thromboembolic complications in patients with COVID-19. Candidate variables were selected based on medical literature and bivariate analyses. Odds ratio (OR) with 95% confidence interval (CI) were estimated using the full model fit and were reported in comparison with the designated reference group. The significance level was defined as α = 0.05.

## Results

Patient characteristics

The study included a total of 276 patients, including 178 (64.5%) men and 98 (35.5%) women. Nearly one-quarter (25.7%) and one-third (33.0%) of patients were aged above 65 years and 51-65 years, respectively. Regarding the duration of symptoms prior to admission, 79 (28.6%) patients had symptom onset of less than one week. Only 22 (8.0%) patients had symptom onset of at least three weeks prior to admission. In total, 53 (19.2%) patients required the use of mechanical ventilation. Only 44 (15.9%) patients were smokers. Most patients were overweight (25.7.%) or obese (48.9%) according to the BMI category (Table 1).

**Table 1:** Patients characteristics

Variables		N	(%)
Age (years)	18–35	50	(18.1)
	36–50	64	(23.2)
	51–65	91	(33.0)
	>65	71	(25.7)
Gender	Male	178	(64.5)
	Female	98	(35.5)
Duration of symptoms prior to admission	≤1 week	79	(28.6)
	1–2 weeks	175	(63.4)
	≥3 weeks	22	(8.0)
Use of mechanical ventilation	Yes	53	(19.2)
	No	223	(80.8)
Smoking status	Smoker	44	(15.9)
	Non-smoker	232	(84.1)
Body mass index category	Underweight	21	(7.6)
	Normal	49	(17.8)
	Overweight	71	(25.7)
	Obese	135	(48.9)

Thromboembolic complications

In total, 64 patients were found to have thromboembolic events, yielding a complication rate of 23.2% among this group. Fifty-one (18.5%) were identified to have pulmonary embolism on the CT scan. Notably, the majority (90.2%) of these pulmonary embolisms were involving the central pulmonary vasculature rather than the segmental and subsegmental branches. Further, 16 (5.8%) patients were identified to have deep venous thrombosis as evident by a Duplex ultrasound examination that was made to investigate the etiology of unilateral lower limb swelling.

Pulmonary radiological findings

**Figure 1:**
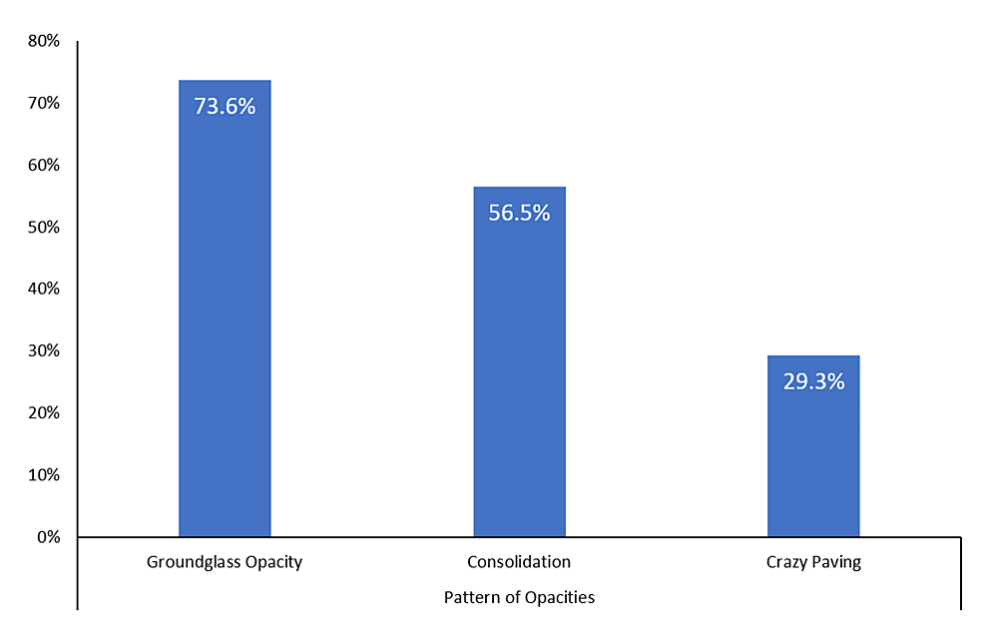
Patterns of the pulmonary opacities on the CT scan of the patients with COVID-19 pneumonia

**Figure 2:**
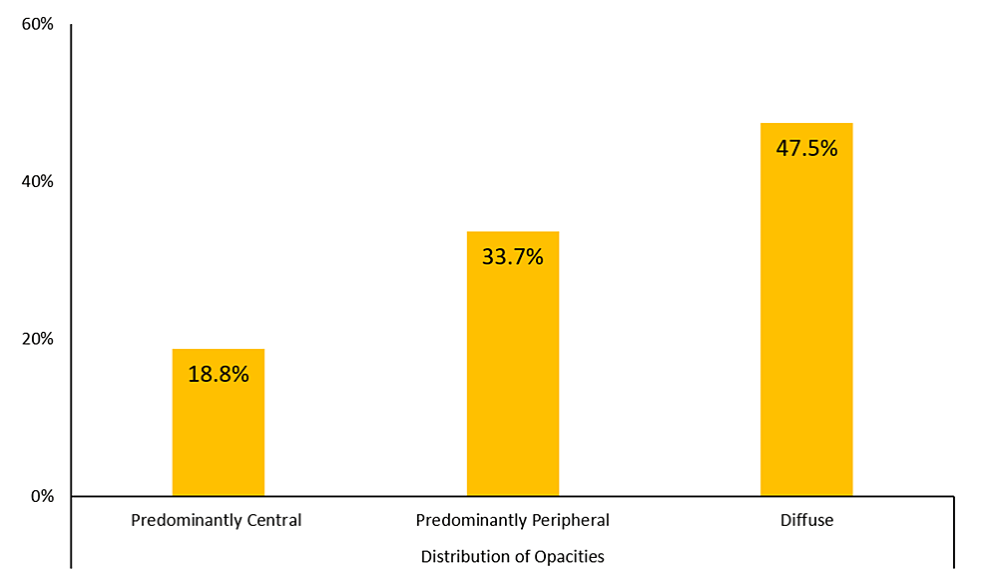
Distribution of opacities on the CT scan of the patients with COVID-19 pneumonia

**Figure 3:**
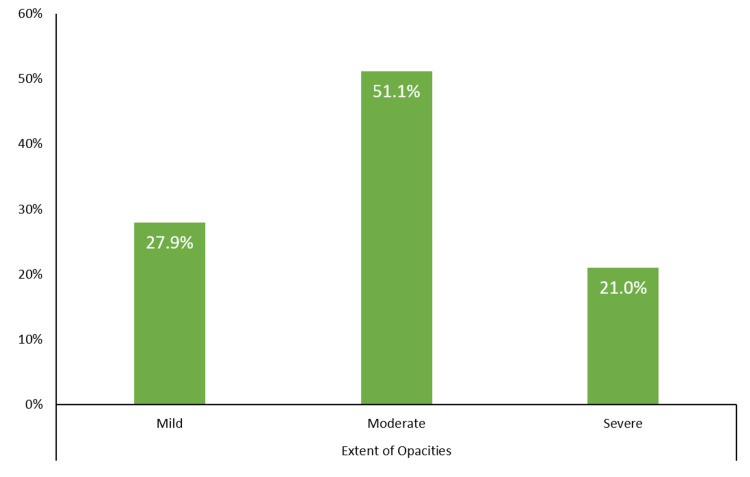
Extent of pulmonary opacities on the CT scan of the patients with COVID-19 pneumonia

The radiological findings of the patients on the CT scans are summarized in Figures 1-3. Ground-glass opacity was the most common pattern of pulmonary opacities, seen in 203 (73.6%) patients. In total, 81 (29.3%) patients had a crazy-paving appearance on the CT scan. Over half (56.5%) of the patients had pulmonary consolidation. Only 52 (18.8%) patients had predominantly central involvement, while the remaining patients had either predominantly peripheral (33.7%) or diffuse (47.5%) involvement. In total, 58 (21.0%) patients were recognized as having severe pulmonary parenchymal involvement. Further, 165 (59.8%) patients had air bronchogram and 85 (30.8%) patients had lymphadenopathy. Pleural effusion and cavitation were uncommon findings, seen in 16 (5.8%) and three (1.1%) patients, respectively.

Patient characteristics associated with thromboembolic events

Table 2 summarizes the bivariate analysis of the association between the patient characteristics and the rate of thromboembolic complications. The patient’s age was significantly associated with the rate of thromboembolic events (P < 0.01). The patients aged 18-35 years were the least likely (8.0%) to develop thromboembolic complications, while patients aged 51-65 years had the highest rate (33.0%) of complications. Female patients had a higher rate of thromboembolic complications compared to their male counterparts (29.6% vs. 19.7%). However, the association was not statistically significant (P = 0.06). Patients who had symptom onset for at least three weeks prior to admission had a higher rate (59.1%) of thromboembolic complications compared to those having the symptoms for ≤one week (13.9%) or one to two weeks (22.9%) (P < 0.01). Further, over half (54.7%) of the patients who had mechanical ventilation developed thromboembolic complications. The smoking status and body mass index were not found to be significantly associated with thromboembolic complications.

**Table 2:** Thromboembolic complications according to CT findings N: Number of patients; CT: Computed tomography; OR: Odds ratio.

Variables		N	(%)	P-values
Pattern of opacities	Ground-glass opacity	51	(25.1)	0.20
	Consolidation	35	(22.4)	0.74
	Crazy-paving	44	(54.3)	<0.01
Distribution of opacities	Predominantly central	14	(26.9)	0.23
	Predominantly peripheral	32	(34.4)	
	Diffuse	18	(13.7)	
Extent of opacities	Mild	7	(9.1)	<0.01
	Moderate	12	(8.5)	
	Severe	45	(77.6)	
Additional features	Air bronchogram	44	(26.7)	0.09
	Pleural effusion	2	(12.5)	0.30
	Lymphadenopathy	19	(22.4)	0.83
	Cavitation	0	(0.0)	0.64

Radiological findings associated with thromboembolic events

The crazy-paving appearance of opacity was significantly associated with the occurrence of thromboembolic events among patients with COVID-19. In particular, 44 (54.3%) patients with crazy-paving appearance developed thromboembolic complications (P < 0.01). However, ground-glass opacity and consolidation were not found to be significantly associated (P > 0.05). Further, there was no significant difference in the rate of thromboembolic events according to the distribution of opacities. However, the extent of opacities was significantly associated with the occurrence of thromboembolic complications as over three-quarters (77.6%) of the patients with severe pulmonary parenchymal involvement developed thromboembolic complications. Other radiological features, including air bronchogram, pleural effusion, lymphadenopathy, and cavitation, were not found to be significantly associated with thromboembolic events (Table 3).

**Table 3:** Thromboembolic complications according to CT findings N: Number of patients; CT: Computed tomography; OR: Odds ratio.

Variables		N	(%)	P-values
Pattern of opacities	Ground-glass opacity	51	(25.1)	0.20
	Consolidation	35	(22.4)	0.74
	Crazy-paving	44	(54.3)	<0.01
Distribution of opacities	Predominantly central	14	(26.9)	0.23
	Predominantly peripheral	32	(34.4)	
	Diffuse	18	(13.7)	
Extent of opacities	Mild	7	(9.1)	<0.01
	Moderate	12	(8.5)	
	Severe	45	(77.6)	
Additional features	Air bronchogram	44	(26.7)	0.09
	Pleural effusion	2	(12.5)	0.30
	Lymphadenopathy	19	(22.4)	0.83
	Cavitation	0	(0.0)	0.64

Multivariable analysis of factors associated with thromboembolic events

Multivariable binary logistic regression analysis was conducted to identify the independent factors associated with thromboembolic complications in patients with COVID-19 pneumonia (Table 4). The model revealed that patients aged 51-65 years (OR = 8.9; 95% CI: 3.0-26.5) and >65 years (OR = 18.7; 95% CI: 7.6-46.1) had a higher likelihood of having thromboembolic complications compared to those aged 18-35 years. Further, patients who had symptom onset of three weeks or more prior to their admission were seven times more likely (OR = 7.2; 95% CI: 1.2-43.0) to have thromboembolic complications compared to those having symptom onset of not more than one week.

**Table 4:** Multivariable analysis of factors associated with thromboembolic complications N: Number of patients; CT: Computed tomography; OR: Odds ratio.

Variables		OR	95% CI	P-values
Age (years)	18–35	Reference group		
	36–50	3.4	[0.8–14.0]	0.09
	51–65	8.9	[3.0–26.5]	<0.01
	>65	18.7	[7.6–46.1]	<0.01
Male gender		0.8	[0.5–1.4]	0.41
Duration of symptoms prior to admission	≤ 1 week	Reference Group		
	1–2 weeks	2.8	[0.9–8.5]	0.07
	≥ 3 weeks	7.2	[1.2–43.0]	0.03
Use of mechanical ventilation		4.2	[0.7–25.6]	0.12
Crazy-paving appearance		14.2	[6.9–29.4]	<0.01
Extent of opacities	Mild	Reference Group		
	Moderate	4.2	[0.9–19.9]	0.07
	Severe	30.6	[9.8–95.5]	0.01

Regarding the radiological features, the crazy-paving appearance of opacity was identified as an independent factor associated with thromboembolic events (OR = 14.2; 95% CI: 6.9-29.4). Further, patients with severe pulmonary parenchymal involvement were 30 times (OR = 30.6; 95% CI: 9.8-95.5) more likely to have thromboembolic complications compared to those having mild involvement.

## Discussion

The study investigated the demographic and radiological characteristics that could serve as independent factors associated with venous thromboembolic disease in patients with COVID-19 pneumonia. The rate of thromboembolic complications in the current cohort is quite high, reaching up to one-fourth of patients. The reported rate of such complications varies significantly in the literature, with the earlier studies showing a relatively larger proportion of patients experiencing thromboembolic complications that may be related to delayed diagnosis and lack of appropriate thromboprophylaxis protocols [9-11]. For example, a multicentric prospective study conducted in 2020 revealed that up to 46% of patients with severe COVID-19 in the intensive care units developed thrombotic complications, mainly pulmonary embolisms [12]. It should be noted, however, that the rate of thromboembolic complications in our study does not necessarily reflect the actual estimates as the inclusion criteria involved only the hospitalized patients who underwent CT scans of the chest.

The study revealed that gender did not influence the risk of venous thromboembolism. In general, the prevalence of thromboembolic complications is higher among men [13]. Further, this gender difference was also demonstrated in a large study involving over 20,000 patients with COVID-19 [14]. In the present study, the gender differences were not observed likely due to the limited sample size.

An interesting finding from the present study is that the duration of symptoms prior to admission was an independent determinant of the thromboembolic events. Such findings may suggest that the thromboembolic complications in COVID-19 tend to develop in the late phases of the disease or that the patients developed such complications and were symptomatic for a while before seeking care. Notably, a growing number of evidence raised the concept of late-onset hematological complications even following the recovery of COVID-19 [15].

The radiological findings in terms of the extent of the disease and the presence of certain opacity patterns, namely, crazy-paving appearance, were independent determinants of the thromboembolic complications. Such findings provided further supporting evidence of the value of CT scans in patients with COVID-19, and the role of the CT is not limited to the evaluation of complications rather it may provide prognostic information about the risk of certain complications. A recent study in 2022 showed that patients with crazy-paving appearances in the chest CT scan were more likely to develop acute kidney injury and had a higher mortality rate [16].

The present study is not without certain limitations. Although it was conducted in one of the largest centers in the region, the study was at a single center with a relatively limited number of patients. Furthermore, the retrospective nature of the study is another important limitation. The study only addressed the thromboembolic events that occurred during the acute phase of the disease, and recent evidence suggested that the thromboembolic complication may have a late onset and may develop even after the recovery from COVID-19.

## Conclusions

The present study reveals a high prevalence of thromboembolic events among the hospitalized patients with COVID-19 who underwent CT chest. Further, the risk of such complications was more common in patients with prolonged onset of symptoms. Also, the radiological findings on the CT scan of the chest can provide crucial prognostic information for patients with COVID-19 in terms of thromboembolic events. Clinicians need to keep a high index of suspicion for pulmonary embolism and deep venous thrombosis when they encounter patients with crazy-paving opacity appearances on CT scans, particularly among patients with severe parenchymal involvement.
